# The causal nexus between diverse smoking statuses, potential therapeutic targets, and NSCLC: insights from Mendelian randomization and mediation analysis

**DOI:** 10.3389/fonc.2024.1438851

**Published:** 2024-11-04

**Authors:** Zhenghua Cao, Shengkun Zhao, Tong Wu, Huan Ding, Zhiyu Tian, Feng Sun, Zhuo Feng, Shaodan Hu, Li Shi

**Affiliations:** ^1^ Graduate School, Changchun University of Chinese Medicine, Changchun, Jilin, China; ^2^ Geriatric Department, Suzhou Hospital of Integrated Traditional Chinese and Western Medicine, Suzhou, Jiangsu, China; ^3^ Respiratory Disease Department, Affiliated Hospital of Changchun University of Chinese Medicine, Changchun, Jilin, China

**Keywords:** different smoking statuses, NSCLC, Mendelian randomization, bioinformatics, mediation analysis

## Abstract

**Objective:**

Lung cancer, the most prevalent malignancy, is typically diagnosed at an advanced stage. Smoking is a pivotal risk factor for NSCLC, yet the impact of various smoking statuses on NSCLC remains unclear. Thus, this study aims to explore whether different smoking statuses can causally influence NSCLC through effects on predictive targets, offering a novel perspective for NSCLC treatment.

**Methods:**

Employing dual-sample MR, MVMR, and TSMR approaches, we assessed the causal relationships between 13 distinct smoking statuses and NSCLC, using predicted potential therapeutic targets as mediators to further elucidate the causal interplay among them.

**Results:**

Among the 13 smoking statuses, current tobacco smoking, exposure to tobacco smoke outside the home, past tobacco smoking, and never smoked demonstrated causal relationships with NSCLC. MVMR analysis reveals that Current tobacco smoking is an independent risk factor for NSCLC. Utilizing NCAPD2, IL11RA, and MLC1 as mediators, IL11RA (22.2%) was found to potentially mediate the relationship between past tobacco smoking and NSCLC.

**Conclusion:**

This study, integrating bioinformatics and MR analysis, identified three potential predictive targets as mediators to investigate the causal relationships between different smoking statuses and NSCLC through potential therapeutic targets, providing new insights for the treatment and prevention of NSCLC.

## 1 Introduction

Lung cancer stands as one of the principal causes of mortality among all diseases globally (1), with both its incidence and mortality rates on the rise (2). In 2022, lung cancer accounted for one-eighth of all global cancer cases, making it the leading cause of cancer-related deaths worldwide. It ranks first in incidence and mortality among men and second among women (3), with non-small cell lung cancer (NSCLC) comprising 80%-85% of all lung cancer cases (4). The primary risk factors for lung cancer include smoking, occupational exposure, air pollution, and genetic susceptibility, each of which can either individually or synergistically elevate the risk of developing lung cancer (5). Recent extensive research has focused on the genetic factors of lung adenocarcinoma, such as comparisons between the lung cancer genes of East Asian and European populations (6), potential therapeutic targets for lung cancer (7, 8), potential susceptibility to lung cancer in Asian populations (9), and the determination of lung cancer susceptibility genes in European, East Asian, and African populations (10). Additionally, rare molecular subtypes of lung cancer have been explored (11). Consequently, further research is imperative to elucidate the underlying mechanisms of lung cancer, enhance our understanding of the disease, and improve early diagnosis and prevention strategies.

The survival rate for advanced lung cancer remains low (12), and there is a definitive causal relationship between the immunological alterations caused by smoking and lung cancer (13). Over 50% of lung cancer cases occur in individuals who have previously smoked (14), with 40% occurring more than 15 years after cessation of smoking (15). With the widespread adoption of health education, an increasing number of lung cancer cases are unrelated to smoking (16). Smoking can increase the risk and mortality rate of lung cancer, establishing a clear causal link (17). However, the specific causal relationships between different smoking statuses and lung cancer remain unclear. Therefore, it is crucial to further explore how various smoking statuses influence lung cancer, which could provide new perspectives for personalized treatment strategies.

Mendelian randomization (MR) allows for the assessment of causal relationships between exposure factors and outcomes from a genetic perspective (18). Single eQTLs may present a risk of biased results and false positives (19), whereas summary data-based Mendelian Randomization (SMR) can explore the pleiotropy between gene expression levels and complex traits (20, 21). Consequently, this study predicts potential etiological and therapeutic targets for NSCLC from a bioinformatics perspective combined with MR analysis. It confirms these potential therapeutic targets through differential analysis in a validation cohort and SMR analysis. By using these potential therapeutic targets as mediators, the study explores whether different smoking statuses can influence NSCLC through the mediation of these targets. Thus, we aim to elucidate the causal relationships between different smoking statuses, potential predictive targets, and NSCLC through MR analysis, thereby offering new therapeutic perspectives for NSCLC.

## 2 Methods

### 2.1 Study design

This study initially employs two-sample MR analysis, using 13 different smoking statuses as exposures. These include Current tobacco smoking, Past tobacco smoking, Ever smoked, Exposure to tobacco smoke outside the home, and Never smoked, as the primary exposures. NSCLC serves as the outcome factor. The research aims to explore the causal relationships and underlying mechanisms between various smoking statuses and NSCLC. After adjusting for the confounding factor of BMI, Multivariable Mendelian Randomization (MVMR) analysis is conducted to ascertain which smoking status independently contributes to the risk of NSCLC. To ensure the credibility of the results, the findings of the MR analysis are validated through the Bayesian Weighted Mendelian Randomization (BWMR) method, confirming the accuracy of the causal relationships established.

Concurrently, we downloaded datasets related to “non-small cell lung cancer” from the GEO database (https://www.ncbi.nlm.nih.gov/geo/) to identify differentially expressed genes associated with NSCLC. Utilizing gene eQTLs as exposure data (https://www.eqtlgen.org/) and NSCLC as outcome data, we conducted MR analyses to identify genes causally linked to NSCLC. By intersecting these genes, we identified co-expressed genes, which were then subjected to enrichment analysis, immune cell infiltration studies, GSEA enrichment analysis, and validation group differential analysis. Additionally, SMR analysis was performed on gene eQTLs and NSCLC to verify the causal relationship of the intersected co-expressed genes.

**Figure 1:**
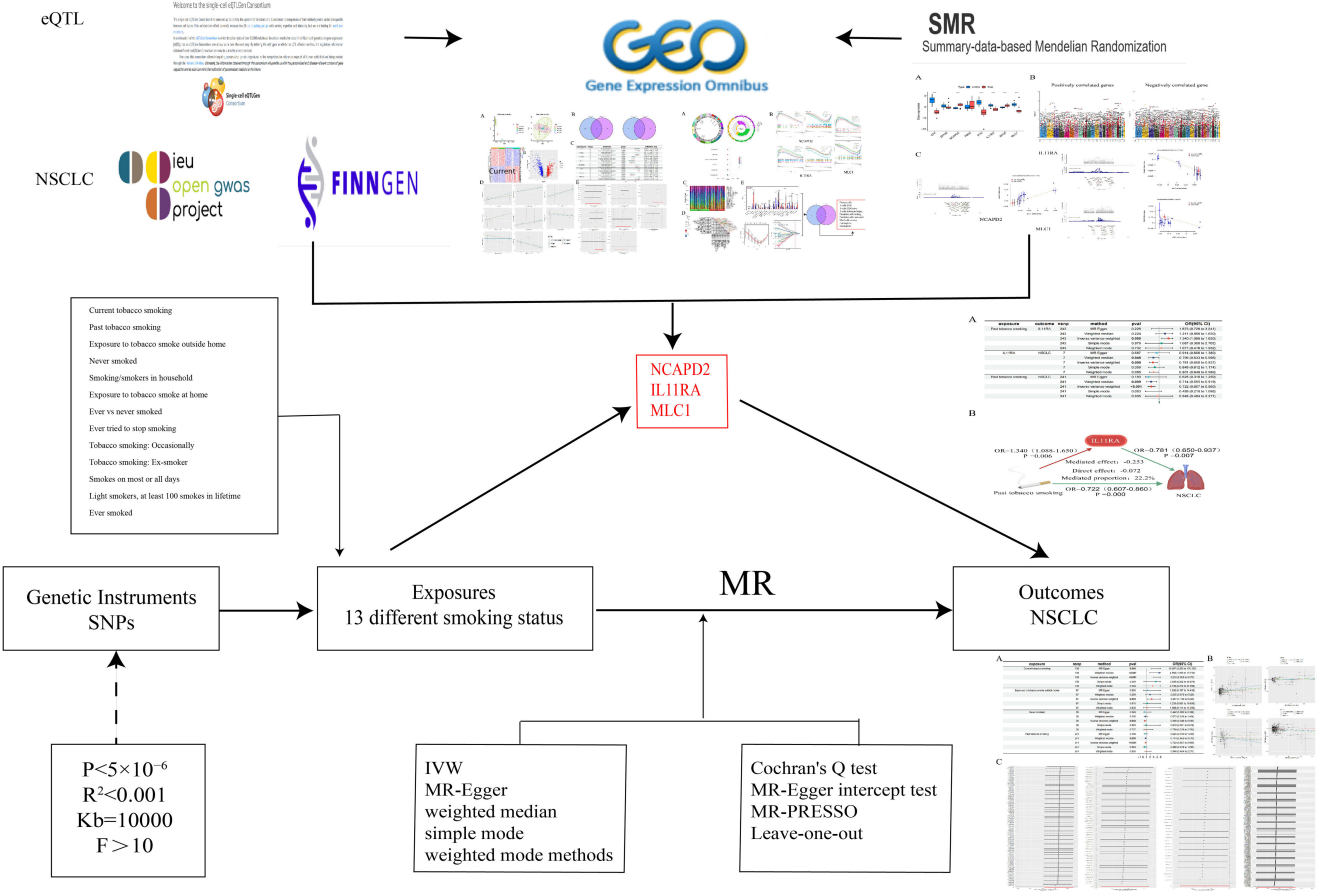
Research approach.

Ultimately, we employ identified potential therapeutic targets for NSCLC as mediators, utilizing two-step MR analysis to further elucidate whether the predicted potential therapeutic targets play a significant mediating role in the causal relationship between various smoking statuses and NSCLC (**Figure 1**).

### 2.2 Data sources

The genetic information for the 13 smoking statuses and BMI is sourced from the GWAS database (https://gwas.mrcieu.ac.uk/), pertaining to a European population. The datasets for differentially expressed genes in the transcriptome are identified by the numbers GSE21933, GSE23066, GSE27262, and GSE118370, while the validation group is denoted by GSE74706. Data on NSCLC are derived from the FinnGen database (https://www.finngen.fi/en) (22), with the specific identifier finngen_R10_C3_LUNG_NONSMALL_EXALLC, also originating from a European cohort. The gene eQTLs data are obtained from peripheral blood of 31,864 European individuals (23, 24) (**Table 1**).

**Table 1:** Sources of data.

Name	ID	Sample Size	SNP
Current tobacco smoking	ukb-b-223	462,434	9,851,867
Past tobacco smoking	ukb-b-2134	424,960	9,851,867
Exposure to tobacco smoke outside home	ukb-b-6244	391,502	9,851,867
Never smoked	ukb-d-22506_114	91,353	13,567,196
Smoking/smokers in household	ukb-b-960	425,516	9,851,867
Exposure to tobacco smoke at home	ukb-b-4462	417,693	9,851,867
Ever vs never smoked	ieu-a-962	74,035	2,455,847
Ever tried to stop smoking	ukb-b-13936	35,910	9,851,867
Tobacco smoking: Occasionally	ukb-d-22506_112	91,353	9,821,991
Tobacco smoking: Ex-smoker	ukb-a-260	83,133	10,894,596
Smokes on most or all days	ukb-d-22506_111	91,353	10,552,527
Light smokers, at least 100 smokes in lifetime	ukb-b-8133	123,894	9,851,867
Ever smoked	ukb-b-20261	461,066	9,851,867
BMI	ukb-b-2303	454,884	9,851,867

### 2.3 Selection of instrumental variables and mendelian randomization analysis

The selection of instrumental variables must satisfy several assumptions (25): the instrumental variables must be closely associated with different smoking statuses, independent of confounding factors in the exposure-outcome relationship, and must influence NSCLC solely through different smoking statuses (26). To ensure their relevance (27), we conduct association analyses for 13 distinct smoking statuses using a threshold of P<5×10^−6^, and for plasma protein eQTLs, a threshold of P<5×10^−8^ is applied. Concurrently, SNPs exhibiting linkage disequilibrium are eliminated using criteria of R^2^<0.001 and a distance of 10,000 Kb (28), followed by the calculation of the F-statistic for the selected SNPs to exclude weak instrumental variables, considering an F-value greater than 10 as indicative of the absence of weak instrumental variables (29, 30).

We employed five methods to assess the causal relationship between the variables: Inverse Variance Weighted (IVW), MR-Egger, Weighted Median, Simple Mode, and Weighted Mode methods, with IVW serving as the primary method (26, 31). A P-value less than 0.05 indicates a causal relationship (32), while the other four methods serve as supplementary approaches (33). Additionally, we utilized the BWMR method to further verify the reliability of the causal relationship. To evaluate the robustness of the causal relationship, a “leave-one-out” sensitivity analysis was conducted (34). Additionally, Cochran’s Q test and the MR-Egger intercept test were utilized to examine pleiotropy and heterogeneity (35, 36), with a P-value greater than 0.05 indicating the absence of both pleiotropy and heterogeneity.

### 2.4 Acquisition of differentially expressed genes and enrichment analysis

We utilized the R programming language to process data from GSE21933, GSE23066, GSE27262, and GSE118370, performing individual data corrections and merging. Using a threshold of P<0.05 and LogFC > 0.585, we filtered differentially expressed genes (DEGs) with the “limma” package, while employing “sva” and PCA to mitigate batch effects. Genes demonstrating a causal relationship between gene eQTLs and NSCLC were extracted and intersected with DEGs to identify co-expressed genes. These intersected genes were then subject to individual MR analyses, along with sensitivity, pleiotropy, and heterogeneity tests, to assess the reliability of the results.

Simultaneously, we conducted GO and KEGG enrichment analyses on the intersected genes using R, aiming to elucidate potential functional pathways and pathogenic mechanisms related to NSCLC. Additionally, GSEA enrichment analysis was employed to explore related functions within the gene expression profiles. We utilized CIBERSORT to analyze the infiltration levels of 22 types of immune cells in NSCLC (37), examining the differential expression of immune cells between two groups. Through Lasso regression, relevant immune cells associated with NSCLC were identified. By intersecting these, core immune cells were determined, further investigating the correlation between intersected genes and immune cells in NSCLC, as well as the regulatory effects of intersected genes on immune cells.

### 2.5 Analysis of differences in the validation group and SMR analysis

We validated the differential expression of intersected genes between the control group and the NSCLC group using data from GSE74706, comparing these findings with the results from MR analysis. Concurrently, we conducted SMR analysis using gene eQTLs data and NSCLC to further ascertain the causal relationship between the intersected genes and NSCLC (20). The SMR software (version 1.3.1) was employed for SMR analysis and HEIDI testing (21), utilizing the default settings of SMR (38). Linkage disequilibrium estimation was based on the European 1000 Genomes as a reference (39). An SMR P-value <0.05 and a HEIDI P-value >0.05 indicate a causal relationship between the exposure and the outcome.

### 2.6 Mediation analysis

Utilizing the TSMR method, we initially calculated the total effect (β_0_) of different smoking statuses on NSCLC, the effect (β_1_) of different smoking statuses on potential therapeutic targets, and the effect (β_2_) of potential therapeutic targets on NSCLC. The mediating effect was computed as β1*β2, and the direct effect was determined by subtracting the mediating effect from the total effect. The mediation proportion was calculated as (β_1_×β_2_)/β_0_ (40), further elucidating whether the predicted potential therapeutic targets play a significant mediating role in the causal relationship between different smoking statuses and NSCLC. All analyses were conducted using R (version 4.3.3).

## 3 Results

### 3.1 The causal relationship between 13 different smoking states and NSCLC

We conducted an association analysis on 13 different smoking statuses, removing linkage disequilibrium and weak instrumental variables, and identified 917 SNPs across these smoking statuses, with the smallest F-statistic being 20.86 and the largest 204.69. Univariate MR analysis supported a causal relationship between NSCLC and various smoking statuses, including Current tobacco smoking, Exposure to tobacco smoke outside the home, Past tobacco smoking, and Never smoked. IVW analysis results indicated a positive correlation between NSCLC and Current tobacco smoking (OR=5.218, 95% CI 2.95-9.373; P=0.000) and Exposure to tobacco smoke outside the home (OR=2.467, 95% CI 1.166-5.220; P=0.018). Conversely, a negative correlation was found with Past tobacco smoking (OR=0.722, 95% CI 0.607-0.860; P=0.000) and Never smoked (OR=0.588, 95% CI 0.348-0.995; P=0.048). Simultaneously, we employed the BWMR method to validate and further ensure the reliability of the causal relationship. Additionally, reverse MR analysis, using NSCLC as the exposure and the four different smoking statuses as outcomes, revealed no reverse causal relationships (P>0.05).

According to the results of the univariate MR analysis, current tobacco smoking, exposure to tobacco smoke outside the home, past tobacco smoking, and never having smoked all exhibit causal relationships with NSCLC. By adjusting for the confounding factor of BMI and conducting MVMR analysis, we found that the causal relationship between current tobacco smoking (OR=6.487, 95% CI 2.670-15.765; P=0.000) and NSCLC persists, affirming that current tobacco smoking is an independent risk factor for NSCLC. In contrast, past tobacco smoking, exposure to tobacco smoke outside the home, and never having smoked are not independent risk factors for NSCLC.

**Figure 2:**
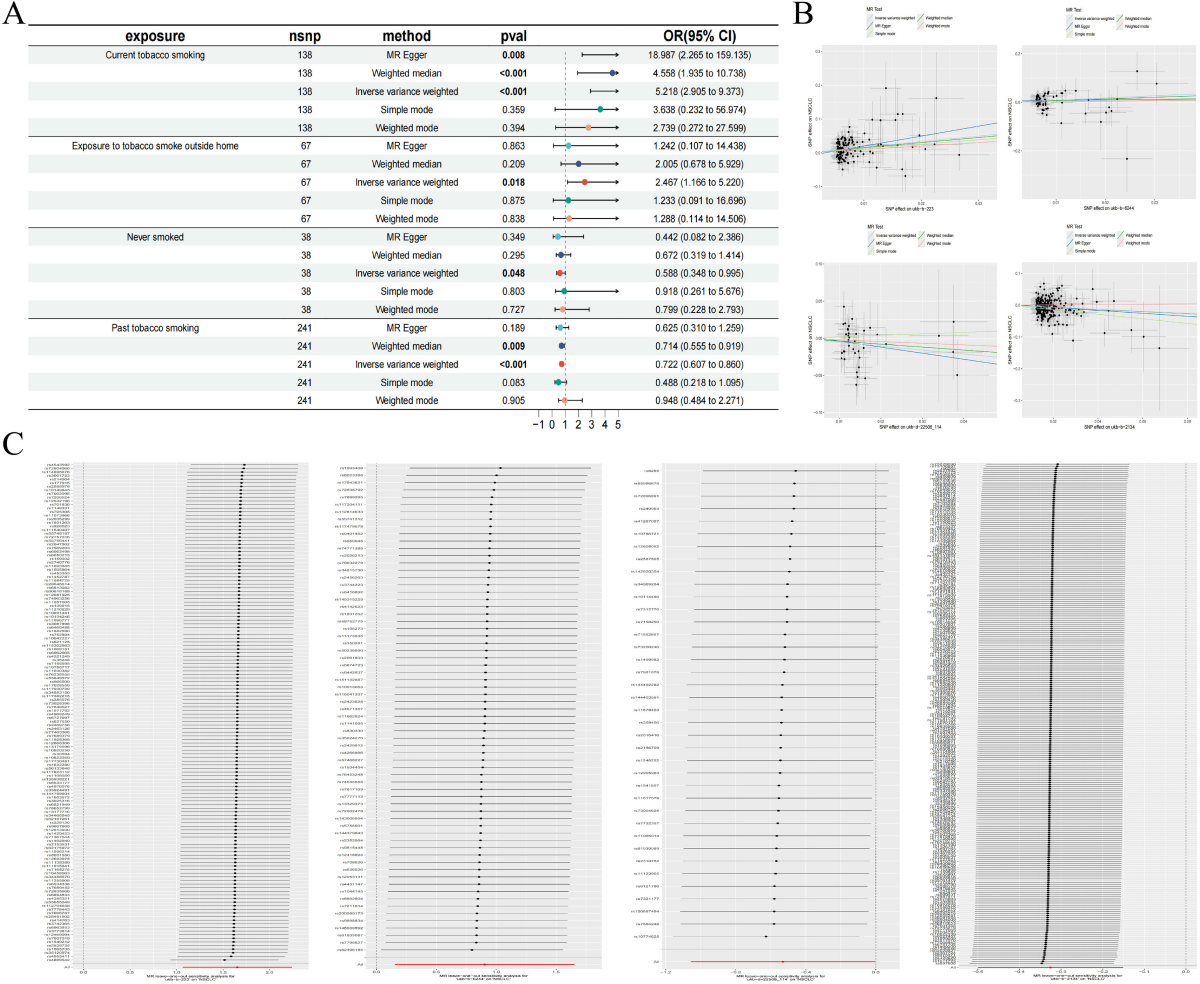
Forest plot of MR analysis for different smoking statuses and NSCLC **(A)**; MR scatter plot for different smoking statuses and NSCLC **(B)**; Results of the leave-one-out sensitivity analysis for different smoking statuses and NSCLC **(C)**.

To evaluate the robustness of our analysis results, we employed Cochran’s Q test, the MR-Egger intercept test, and MR-PRESSO to assess pleiotropy and heterogeneity. No evidence of pleiotropy or heterogeneity was detected (P>0.05). Furthermore, a leave-one-out sensitivity analysis indicated that the exclusion of any single SNP would not significantly affect the estimates of causal relationships, suggesting that the MR analysis results are robust (**Tables 2**, **3**, **Figure 2**).

**Table 2:** MR and sensitivity analysis of different smoking statuses and NSCLC.

Exposure	Method	snp	Beta	Se	P	BWMR	Pleiotropy Test		Heterogeneity Test	
							MR- PRESSO	MR-Egger intercept	IVW Q	MR- Egger Q
ukb-b-2134	IVW	241	-0.325	0.088	0.000	0.000	0.194	0.002 (P=0.676)	258.931 (P=0.191)	258.742 (P=0.181)
ukb-d-22506_114	IVW	38	-0.530	0.268	0.048	0.049	0.615	0.004 (P=0.728)	34.457 (P=0.588)	34.334 (P=0.547)
ukb-b-6244	IVW	67	0.902	0.382	0.018	0.018	0.536	0.005 (P=0.566)	60.090 (P=0.681)	59.758 (P=0.660)
ukb-b-223	IVW	138	1.652	0.298	0.000	0.000	0.345	-0.009 (P=0.217)	143.147 (P=0.342)	141.551 (P=0.354)

**Table 3:** The MVMR Analysis of Four Smoking States and NSCLC.

Exposure	Outcome	Beta	Se	P	OR	95%CI
ukb-b-2134	NSCLC	-0.127	0.198	0.522	0.880	0.596-1.300
ukb-d-22506_114		0.279	0.397	0.481	1.322	0.607-2.881
ukb-b-6244		-0.133	0.518	0.797	0.875	0.316-2.420
ukb-b-223		1.869	0.452	0.000	6.487	2.670-15.765
BMI		0.075	0.072	0.299	1.078	0.935-1.244

### 3.2 Acquisition of DEGs in NSCLC and MR analysis of gene eQTLs and NSCLC

We retrieved transcriptomic data from the GEO database, specifically datasets GSE21933, GSE23066, GSE27262, and GSE118370, which included samples from 57 normal lung tissues and 57 NSCLC lung tissues. Each dataset was corrected and merged to eliminate batch effects, resulting in the identification of 740 differential genes, comprising 343 upregulated and 397 downregulated genes. Concurrently, we conducted an association analysis on gene eQTLs, removing linkage disequilibrium and weak instrumental variables, and identified 19,739 SNPs associated with gene eQTLs, with the smallest F-statistic being 29.71 and the largest 14,522.86. MR analysis revealed causal relationships between 163 gene eQTLs and NSCLC, with 81 showing a negative correlation and 82 a positive correlation.

**Figure 3:**
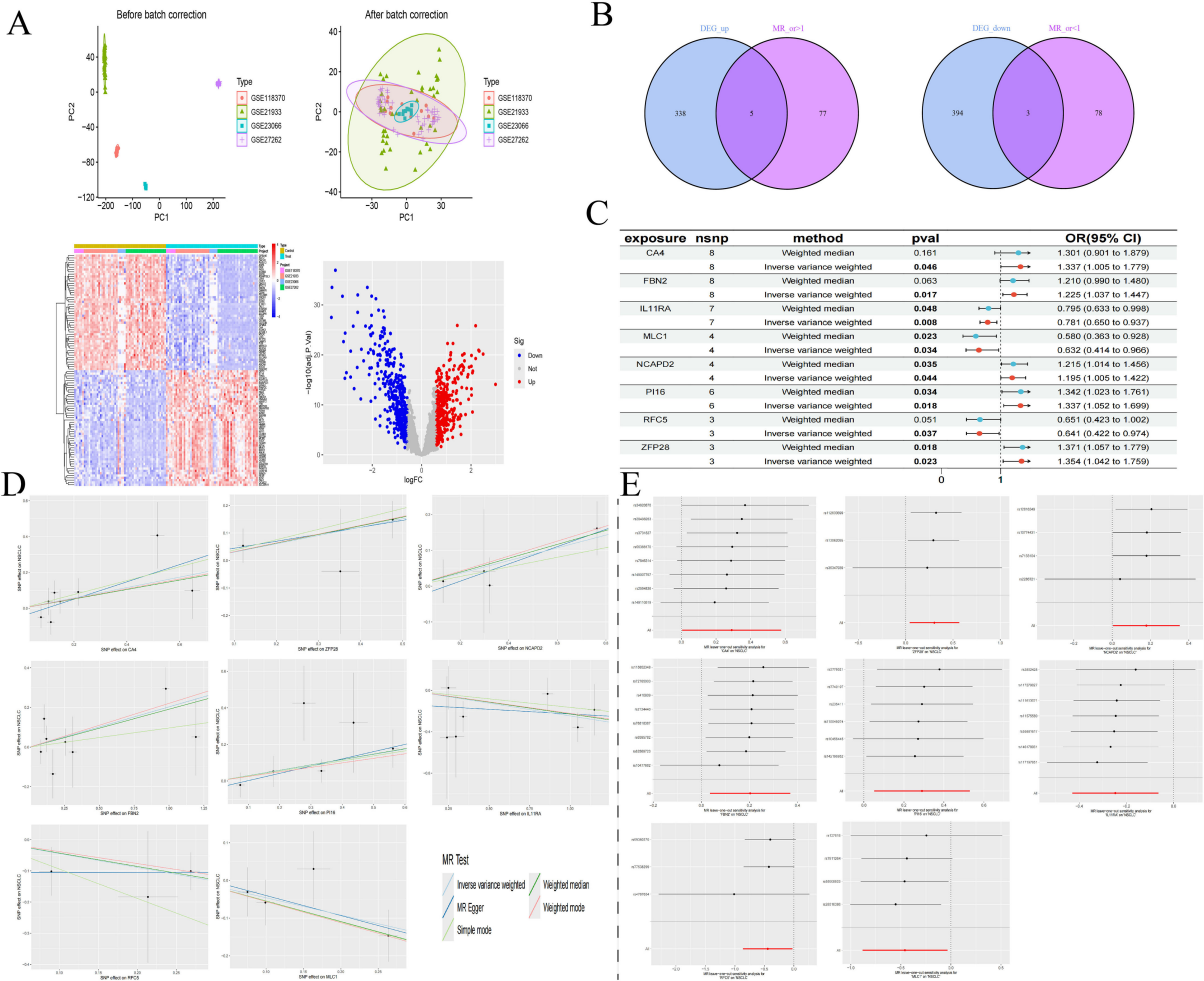
DEGs of NSCLC **(A)**; Intersection of co-expressed genes **(B)**; MR forest plot of co-expressed genes **(C)**; MR scatter plot of co-expressed genes **(D)**; Results of the leave-one-out sensitivity analysis of co-expressed genes **(E)**.

In our MR analysis, we identified 163 genes with causal relationships and intersected these with the 740 genes identified as DEGs, revealing co-expressed genes related to NSCLC. Among these, five upregulated genes were identified: CA4 (OR=1.337, 95% CI 1.005-1.779; P=0.046), ZFP28 (OR=1.355, 95% CI 1.042-1.759; P=0.023), NCAPD2 (OR=1.195, 95% CI 1.005-1.422; P=0.044), FBN2 (OR=1.225, 95% CI 1.037-1.447; P=0.017), and PI16 (OR=1.337, 95% CI 1.052-1.699; P=0.018). Additionally, three downregulated genes were identified: IL11RA (OR=0.781, 95% CI 0.650-0.937; P=0.007), RFC5 (OR=0.641, 95% CI 0.422-0.974; P=0.037), and MLC1 (OR=0.632, 95% CI 0.414-0.966; P=0.034). Simultaneously, we employed the BWMR method to validate and further ensure the reliability of the causal relationships identified. To assess the robustness of our analytical results, we employed Cochran’s Q test and the MR-Egger intercept test to examine pleiotropy and heterogeneity, detecting neither (P > 0.05). The leave-one-out analysis indicated that the exclusion of any single SNP would not significantly affect the estimates of causal association, suggesting that the MR analysis is robust (**Table 4**, **Figure 3**).

**Table 4:** MR and sensitivity analysis of co-expressed genes with NSCLC.

Exposure	Method	snp	Beta	Se	P	BWMR	Pleiotropy Test	Heterogeneity Test	
							MR-Egger intercept	IVW Q	MR-Egger Q
CA4	IVW	8	0.290	0.145	0.046	0.047	-0.044 (P=0.361)	6.857 (P=0.443)	5.883 (P=0.436)
ZFP28	IVW	3	0.303	0.133	0.023	0.023	0.019 (P=0.860)	0.494 (P=0.780)	0.445 (P=0.504)
NCAPD2	IVW	4	0.178	0.088	0.044	0.044	-0.034 (P=0.656)	0.647 (P=0.885)	0.380 (P=0.826)
FBN2	IVW	8	0.202	0.085	0.017	0.014	0.0001 (P=0.997)	7.404 (P=0.388)	7.404 (P=0.285)
PI16	IVW	6	0.290	0.122	0.018	0.018	-0.037 (P=0.619)	4.209 (P=0.519)	3.919 (P=0.416)
IL11RA	IVW	7	-0.247	0.093	0.007	0.010	-0.138 (P=0.439)	4.801 (P=0.569)	4.096 (P=0.535)
RFC5	IVW	3	-0.444	0.213	0.037	0.038	-0.104 (P=0.544)	0.900 (P=0.637)	0.144 (P=0.703)
MLC1	IVW	4	-0.458	0.216	0.034	0.034	0.013 (P=0.875)	1.311 (P=0.726)	1.280 (P=0.527)

### 3.3 Enrichment analysis and immune infiltration of co-expressed genes

We visualized the co-expressed genes to elucidate their distribution in staining. Subsequently, we conducted GO and KEGG enrichment analyses on these genes to explore their potential roles. The GO enrichment analysis revealed that the co-expressed genes predominantly affect biological functions such as cytokine receptor activity and hormone activity, through processes occurring at the extracellular side of the plasma membrane, microfibrils, plasma membrane rafts, caveolae, and involving caveolin-mediated endocytosis, extracellular regulation of signal transduction, and regulation of response to osmotic stress. The KEGG enrichment analysis focused on nucleotide excision repair, mismatch repair, DNA replication, and base excision repair. Additionally, single-gene GSEA analysis was performed on the co-expressed genes to investigate the biological functions or pathway activity levels in the high expression group. For instance, IL11RA is associated with a defense response to other organisms, innate immune response, and vesicle membrane in low expression, and with regulation of cell differentiation and metanephros development in high expression. MLC1, in high expression, is associated with circulatory system processes, multicellular organism processes, and sodium ion transport, while in low expression, it is linked to chromosome organization and chromosomal regions.

**Figure 4:**
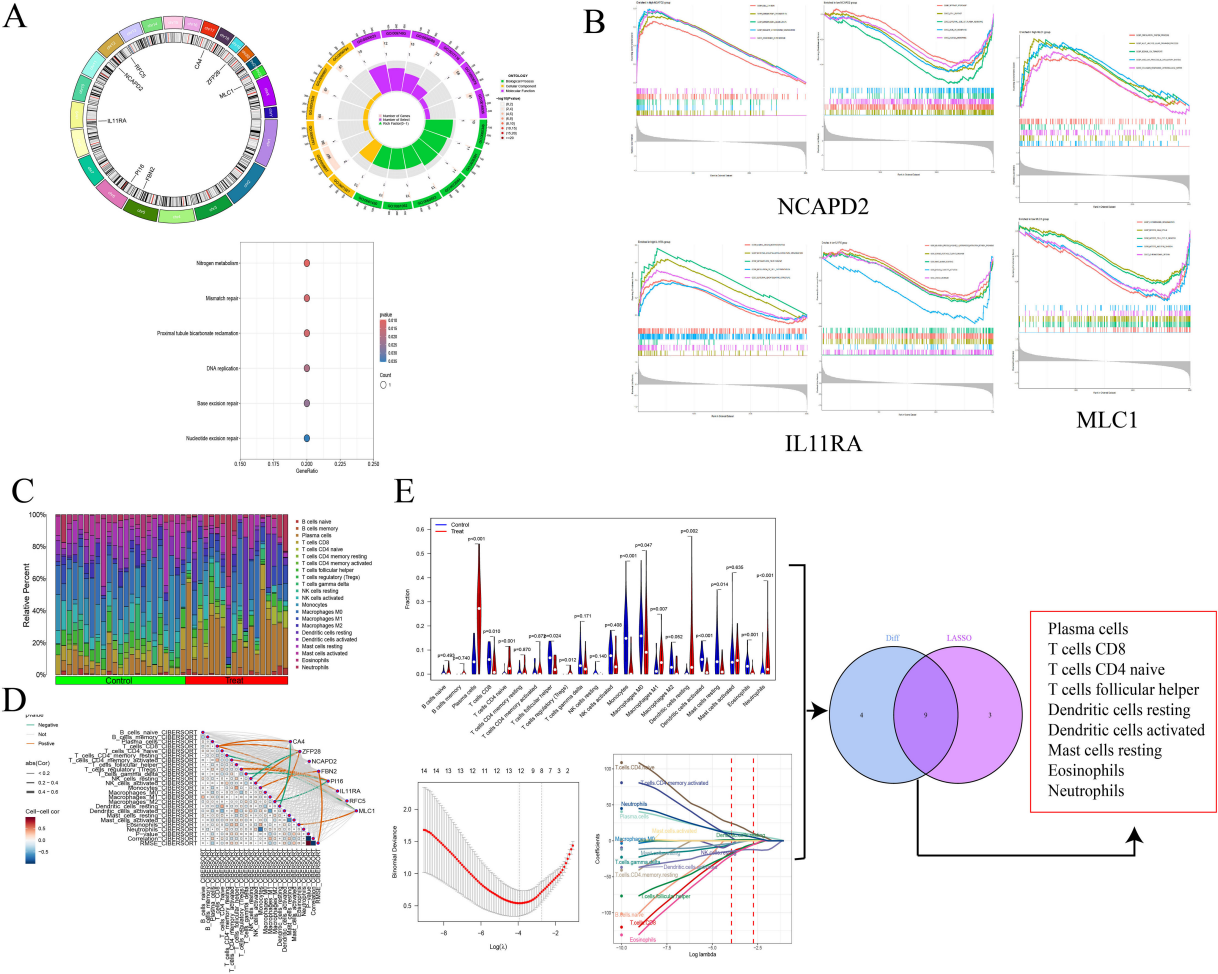
Circos plot and enrichment analysis of co-expressed genes **(A)**; GAEA enrichment analysis for NCAPD2, IL11RA, and MLC1 **(B)**; Immune infiltration analysis **(C)**; Correlation between genes and immune cells **(D)**; Screening of core immune cells **(E)**.

We utilized the CIBERSORT algorithm to infer the characteristics of immune cells and explore the correlation between NSCLC co-expressed genes and immune cells. Compared to the control group, differences were observed in 13 types of immune cells (P < 0.05). In NSCLC samples, the proportions of Plasma cells, T cells CD8, T cells CD4 naive, T cells regulatory (Tregs), Macrophages M0, Macrophages M1, Dendritic cells resting, and Neutrophils were elevated, whereas the proportions of T cells follicular helper, Dendritic cells activated, Monocytes, Mast cells resting, and Eosinophils were reduced. Through Lasso regression, 12 types of immune cells were selected, including Plasma cells, T cells CD8, T cells CD4 naive, T cells CD4 memory activated, T cells follicular helper, and Eosinophils. An intersection of these identified nine core immune cells: Plasma cells, T cells CD8, T cells CD4 naive, and Eosinophils, among others, further suggesting a significant correlation with NSCLC. Exploring the correlation between co-expressed genes and immune cells, it was found that ZFP28 and B cells naive were negatively correlated, as were ZFP28 and Macrophages M2; NCAPD2 was negatively correlated with Dendritic cells resting and Mast cells activated; FBN2 was negatively correlated with T cells CD4 memory activated and Monocytes; PI16 was positively correlated with Dendritic cells activated, MLC1 with T cells CD4 naive; IL11RA was positively correlated with T cells CD8 and negatively with Macrophages M0; CA4 and RFC5 showed no correlation with immune cells (**Figure 4**).

### 3.4 Differential analysis in the validation cohort and SMR analysis

We further validated the co-expressed genes using data from the validation cohort GSE74706, which included 18 samples of normal lung tissue and 18 samples of NSCLC lung tissue. We observed that CA4, PI16, IL11RA, and MLC1 were highly expressed in the control group, while NCAPD2 and RFC5 were expressed at lower levels (P < 0.001). Compared to our MR analysis, the findings for IL11RA, NCAPD2, and MLC1 were consistent with the MR results, further affirming the potential of these three genes in regulating NSCLC.

**Figure 5:**
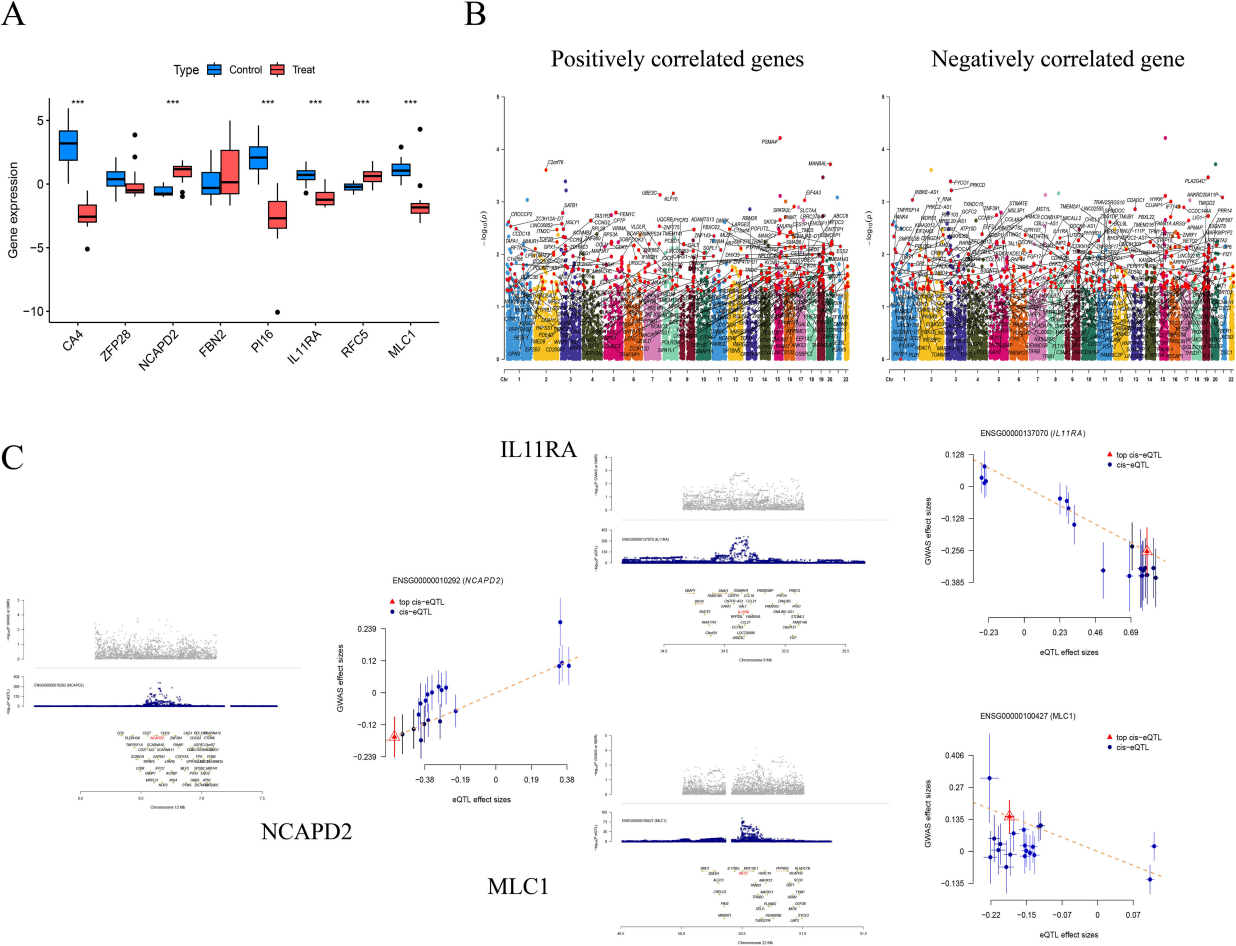
Differential analysis in the validation group (***P<0.001) **(A)**; Manhattan Plots for SMR Analysis (Positive and Negative Correlations) **(B)**; Scatter Plots of Single Genes as Potential Therapeutic Targets for NSCLC (MLC1 and IL11RA are negatively correlated, while NCAPD2 is positively correlated) **(C)**.

We employed SMR to further ascertain the causal relationships between gene eQTLs and NSCLC, revealing that 552 genes have a causal association with NSCLC, with 287 showing a negative correlation and 265 a positive correlation. Among the eight co-expressed genes related to NSCLC, IL11RA, NCAPD2, and MLC1 passed both the SMR analysis and HEIDI tests (P_SMR_ < 0.05 and P_HEIDI_ > 0.05), further substantiating the potential of IL11RA, NCAPD2, and MLC1 as prospective therapeutic targets for NSCLC (**Table 5**, **Figure 5**).

**Table 5:** SMR Analysis of Co-expressed Genes with NSCLC.

Gene	TOP_SNP_	B_GWAS_	SE_GWAS_	P_GWAS_	B_eQTL_	SE_eQTL_	P_eQTL_	B_SMR_	SE_SMR_	P_SMR_	P_HEIDI_
IL11RA	rs2070074	-0.259	0.095	0.006	0.788	0.012	0.000	-0.329	0.120	0.006	0.430
MLC1	rs137919	0.146	0.068	0.033	-0.180	0.009	5.05E-87	-0.811	0.383	0.034	0.062
NCAPD2	rs714775	-0.1653	0.074	0.027	-0.546	0.009	0.000	0.302	0.136	0.027	0.169

### 3.5 Mediation analysis

**Figure 6:**
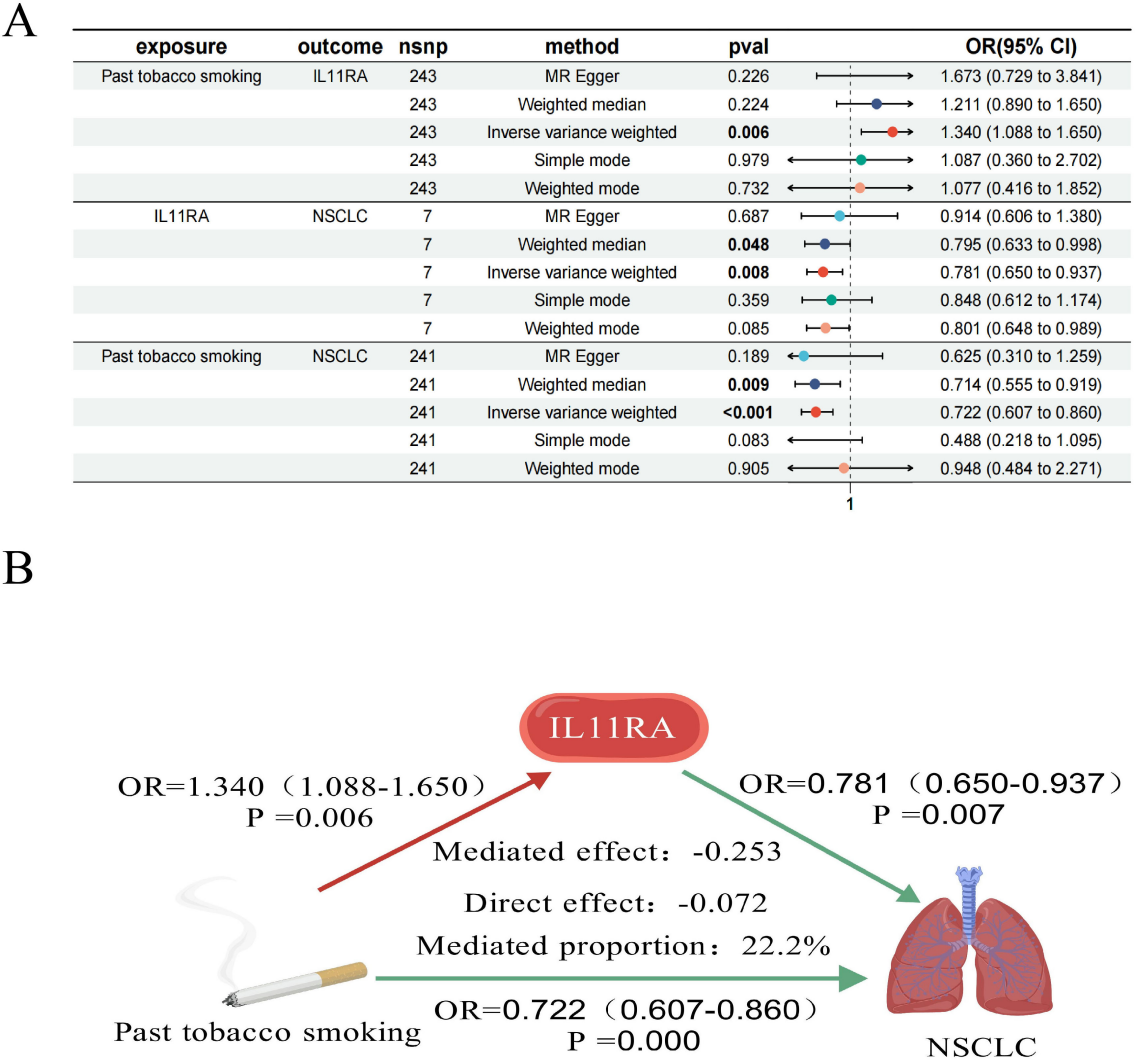
Forest plot of past tobacco smoking, IL11RA, and NSCLC **(A)**; IL11RA Mediates the Causal Relationship between Past Tobacco Smoking and NSCLC (Red Indicates Risk Factors, Green Indicates Protective Factors) **(B)**.

We conducted a mediation analysis focusing on NCAPD2, IL11RA, and MLC1 as potential therapeutic targets for NSCLC, to further explore whether these targets mediate the impact of various smoking statuses on NSCLC. Our aim was to elucidate the proportion of the causal effects of four distinct smoking statuses on NSCLC mediated by these three predicted potential targets. We discovered that only IL11RA mediated the impact of past tobacco smoking on NSCLC, with a mediation effect of -0.253, a direct effect of -0.072, and a mediation proportion of 22.2% (**Figure 6**).

## 4 Discussion

In our study, univariate MR analysis revealed causal relationships between current tobacco smoking, exposure to tobacco smoke outside the home, past tobacco smoking, never smoked, and NSCLC. After adjusting for BMI, further MVMR analysis identified current tobacco smoking as an independent risk factor for NSCLC. Utilizing bioinformatics, MR analysis, and SMR analysis, we identified three potential therapeutic targets for NSCLC (NCAPD2, IL11RA, and MLC1). Further investigation using these three potential targets as mediators revealed that IL11RA might partially mediate the causal relationship between past tobacco smoking and NSCLC. Our findings offer a novel perspective for the treatment of NSCLC.

Smoking is the principal cause of lung cancer (41), with an increased mortality rate associated with smoking (42). Smoking drives the aggregation of pro-inflammatory macrophages (43), induces the expression of CCNA (44), accumulates M2-TAMs (45), and causes oxidative damage (46), thereby promoting the development of NSCLC from multiple angles. This aligns with our research, which genetically demonstrates that the longer the duration of current tobacco smoking, the higher the risk of NSCLC. Compared to active smoking, the mechanisms of passive smoking remain unclear (47); however, systematic reviews have found that exposure to secondhand smoke at home significantly increases the risk of lung cancer (48). Secondhand smoke affects lung function and the production of inflammatory cytokines (49), inducing inflammation and impairing immunity (50). Our genetic research confirms that exposure to tobacco smoke outside the home increases the risk of NSCLC, suggesting that encouraging family members to quit smoking and avoid secondhand smoke is one of the measures to reduce the risk of NSCLC. Numerous studies have found that quitting smoking earlier and for longer durations can reduce the mortality rate of NSCLC (51), and quitting smoking, whether before or after diagnosis, is beneficial for the survival of NSCLC patients (52, 53), enhancing overall survival (54). However, there is no significant difference in survival between smokers and non-smokers with NSCLC (55). Our MR analysis found a negative correlation between past tobacco smoking and NSCLC, consistent with most observational studies and systematic reviews. The incidence of NSCLC in never-smokers is increasing (56), with variations due to geography, environment, gender, and ethnicity (57). Our study indicates a causal relationship between never smoking and NSCLC, further emphasizing the genetic susceptibility to NSCLC.

Through bioinformatics and MR analysis, we have predicted eight genes potentially relevant for the prevention and treatment of NSCLC, with five showing positive correlations (CA4, ZFP28, NCAPD2, FBN2, and PI16) and three exhibiting negative correlations (IL11RA, RFC5, and MLC1). Cytokine receptor activity can augment the chemotherapeutic immune response in NSCLC (58), playing a synergistic role in combating tumors (59). There is a notable association between hormone activity and NSCLC (60); women receiving estrogen therapy have a lower mortality rate from lung cancer compared to men (61), and estrogen can influence the immune suppression response in NSCLC (62), inhibiting the growth of lung cancer cells through hormone activity (63). Nucleotide excision repair can fix mutated DNA bases, serving as a marker for early lung cancer cells (64). We conducted an immune infiltration analysis, identifying nine core immune cells in NSCLC through differential immune cells and Lasso regression, and explored the co-expression of genes and their correlation with immune cells. Plasma cells, which produce specific antibodies to initiate an immune response, are prognostically relevant to NSCLC (65) and contribute significantly to the efficacy of PD-1 blockade in NSCLC treatment (66). Enhancing the immune infiltration of T cells CD8 can improve the therapeutic efficacy against NSCLC (67), promoting anti-tumor activity (68). Eosinophils can predict the response and prognosis of NSCLC treatment (69) and may serve as predictive biomarkers (70). Neutrophils can be linked to new therapeutic approaches in NSCLC (71).

Further validation groups and SMR have identified three potential targets for the prevention and treatment of NSCLC: NCAPD2, IL11RA, and MLC1. NCAPD2, located on chromosome 12, regulates the structure and separation of chromosomes (72) and heterochromatin recombination (73), affecting cellular function abnormalities and playing a role in tumor development. Downregulation of NCAPD2 can inhibit tumor growth *in vivo (* 74), promote the release of pro-inflammatory factors (75), enhance cell cycle progression and invasive capabilities (76), regulate autophagy (77), and serve as a prognostic biomarker in various cancers (78). It is often overexpressed in pan-cancer settings, associated with poor outcomes (79).MLC1, located on chromosome 22, is typically associated with the white matter of the central nervous system and is highly expressed in vascular astrocytes (80). It determines the proliferation and invasive state of brain cancer glioma cells (81). MLC1 expression is negatively correlated with tumor metastasis and has an anti-tumor effect (82). MLC1 enhances the sensitivity of cells to apoptosis, improving the effectiveness of NSCLC treatment (83).IL11RA, located on chromosome 9, initiates signaling pathways that affect cell growth, proliferation, and differentiation. IL11RA interacts with IL11 (84) and can regulate inflammatory responses, bone metabolism, and tumor development through the IL-11 signaling pathway (85). IL11RA is associated with T cells CD8 and can inhibit tumor growth (86), enhance drug resistance (87), and has potential immunomodulatory effects (88), improving survival outcomes (89). Research on the relationship between IL11RA, past tobacco smoking, and NSCLC is limited. However, through MR analysis, bioinformatics, and mediation analysis, we discovered that past tobacco smoking could exert a mediating effect on NSCLC through the mediation of IL11RA. This provides a fresh perspective for the treatment of NSCLC.

## 5 Conclusion

This study employs bioinformatics and MR analysis to identify three potential predictive targets, using them as mediators to explore the causal relationship between past tobacco smoking in various smoking statuses and NSCLC through the potential therapeutic target IL11RA. This approach offers a novel perspective for the treatment and prevention of NSCLC.

## Data Availability

The original contributions presented in the study are included in the article/supplementary material. Further inquiries can be directed to the corresponding authors.
